# Flow Cytometry Analyses of Meningioma Immune Cell Composition Using a Short, Optimized Digestion Protocol

**DOI:** 10.3390/cancers16233942

**Published:** 2024-11-25

**Authors:** Gillian Dao Nyesiga, Jeppe Lohfert Haslund-Vinding, Josephine Budde, Josefine Føns Lange, Nadja Blum, Kotryna Dukstaite, Lars Ohlsson, Tiit Mathiesen, Anders Woetmann, Frederik Vilhardt

**Affiliations:** 1 Department of Biomedical Sciences, Faculty of Health and Society, Malmö University, 205 06 Malmo, Sweden; lars.ohlsson@mau.se; 2 Department of Neurosurgery, Copenhagen University Hospital—Rigshospitalet, 2100 Copenhagen, Denmark; jeppe.lohfert.haslund-vinding.01@regionh.dk (J.L.H.-V.); josephine.bjoern.budde.01@regionh.dk (J.B.); josefine.lange@regionh.dk (J.F.L.); nadjablum@sund.ku.dk (N.B.); tiit.illimar.mathiesen@regionh.dk (T.M.); 3 Department of Cellular and Molecular Medicine, Faculty of Health and Medical Sciences, University of Copenhagen, 2200 Copenhagen, Denmark; kotryna@sund.ku.dk (K.D.); vilhardt@sund.ku.dk (F.V.); 4 Department of Clinical Medicine, Faculty of Health and Medical Sciences SUND, University of Copenhagen, 2200 Copenhagen, Denmark; 5 Department of Clinical Neuroscience, Karolinska Institutet, 171 76 Stockholm, Sweden; 6 LEO Foundation Skin Immunology Research Center, Department of Immunology and Microbiology, Faculty of Health and Medical Sciences, University of Copenhagen, 2200 Copenhagen, Denmark; awoetmann@sund.ku.dk

**Keywords:** meningioma, immune cells, macrophages, lymphocytes, tumor-infiltrating cells, brain tumor, T cells, TIM-3, tumor digestion, single-cell suspension

## Abstract

Background: Current challenges in meningioma treatment, including post-surgical complications and cognitive impairments, highlight the need for new treatment alternatives. Immunological interventions have shown promise. However, there is a knowledge gap in characterizing infiltrating immune cells in meningioma and their interplay. Further studies on immune cells in single-cell suspensions from digested meningioma tissues could identify targetable mechanisms for non-surgical treatment options with fewer side effects. This study aimed to optimize a protocol for faster digestion of meningioma tissues into viable single-cell suspensions and to identify infiltrating immune cell populations. Methods: We modified a commercial kit intended for whole skin dissociation to digest resected meningioma tissues into viable single-cell suspensions. Tumor-infiltrating immune cell populations were characterized using flow cytometry. Results: Flow cytometry analyses revealed that the digested tissue was composed of viable immune cells, including predominantly CD14^+^ macrophages and CD3^+^ T cells, with minor populations of CD56^+^ NK cells and CD19^+^ B cells. In both of the two patient samples tested, half of the tumor-associated macrophages were TIM-3^+^, with a small proportion co-expressing CD83. Women were more likely to have a lower proportion of immune cells, B cells, and NK cells. Female patients with a high proportion of immune cells had a higher proportion of macrophages. Conclusion: We successfully optimized a protocol for generating single-cell suspensions with viable immune cells from meningioma tissues, revealing infiltrating antigen-presenting cells with an immunosuppressive phenotype, and lymphocytes. This short protocol allows advanced analyses of tumor-infiltrating cells using techniques such as single-cell RNA sequencing and flow cytometry, which require live, dissociated cells.

## Simple Summary

Due to post-treatment side effects such as post-surgical complications and cognitive impairments in meningioma patients, new alternatives are needed, e.g., immunotherapy. However, the interplay between the tumor and the infiltrating immune cells is not fully understood. By modifying a commercial tissue digestion kit, we aimed to provide a shorter protocol for digesting meningioma tissues into viable single-cell suspensions and to investigate the tumor-infiltrating immune cells. Flow cytometry analyses revealed single-cell suspensions of meningiomas containing viable immune cells, predominantly CD14^+^ macrophages and CD8+ T cells, with smaller proportions of B cells and NK cells. We successfully optimized a shorter protocol for generating single-cell suspensions with viable immune cells from meningioma tissues, allowing for advanced analyses of tumor-infiltrating cells using techniques such as single-cell RNA sequencing and flow cytometry, which are not applicable to intact tissue or dead cells.

## 1. Introduction

Meningioma is the most common primary central nervous system tumor and accounts for up to 41% of all primary brain tumors [1]. These brain tumors are thought to arise from arachnoid cap cells adjacent to the dura mater, one of the protective layers of the brain [2]. Although the majority of meningiomas are classified as slow-growing, benign World Health Organization (WHO) grade I tumors, the pressure from the growing tumor may give rise to other symptoms that can range from mild to severe, for example, from headaches to seizures (National Cancer Institute, USA). Even benign meningiomas can cause significant neurological, psychological, and oncological morbidity regardless of whether they are operated on or left untreated [3,4,5,6,7,8,9].

A significant unmet medical need remains in the treatment of meningiomas. Current interventions are limited to craniotomy for surgical resection and to radiotherapy. Patients suffer from surgical complications [10] and experience cognitive impairment after surgery or radiotherapy, including depression and anxiety, or deficits of attention, orientation, memory, and language [11]. An even greater unsolved issue is meningiomas that recur or progress despite treatment [12]. Therefore, non-surgical treatments that would provide a biological approach are sought-after.

Despite the success of immune checkpoint inhibitors (ICIs) and cell-based immunotherapies in solid tumors [13,14], their effectiveness in meningiomas remains underexplored. The microenvironment in meningiomas appears to be immunosuppressive, in particular for the more aggressive grade 2 and 3 tumors, but its descriptions remain incomplete [15,16,17]. Immune checkpoint inhibitor therapy targeting PD-1 showed promising preliminary results for recurrent high-grade meningioma when administered in a cohort of eight patients with recurrent meningioma following surgical resection and radiotherapy [18]. In addition, two phase II clinical trials targeting PD-1 for the treatment of meningioma showed contrasting results: one reported prolonged progression-free survival [19], while the other reported no improved progression-free survival of 6 months [20]. Contextualization of these findings is difficult as long as the immunological crosstalk in the tumor microenvironment remains unknown. Although research on cell populations in meningioma has been performed, a crucial gap still exists in comprehending the immune cell composition and the interactions between these cells in meningiomas.

Although significant progress has been made in enzymatic digestion of meningiomas into single cells [21], further refinement of the protocol could enhance cell suspension quality and reduce digestion time, improving overall efficiency of laboratory processes that may follow. Since patient material is difficult to obtain, most work using single cells in meningioma is with cell lines and not donor-specific primary cells that would provide a more authentic scenario [22]. Single-cell assays with cells obtained from original tissues are preferable for exploring the interaction between the tumor microenvironment and immune cells. Different cell populations can be isolated from single-cell suspensions of digested primary meningioma tissue, enabling cell-based culture assays to investigate the crosstalk and contribution to immune evasion of each population.

Thus, this study aimed to provide a shorter, optimized protocol for the digestion of meningioma tissue into a single-cell suspension with viable immune cells suitable for downstream analyses of tumor-infiltrating immune cell populations. This digestion protocol enables researchers to use assays and technologies, which are otherwise not feasible due to poor viability or a limited number of cells, for the analysis of immunosuppressive networks and exploration of targetable mechanisms for immunotherapy.

Here, we report a short protocol for the digestion of meningioma tissue into a single-cell suspension that contains viable immune cells that comprise of predominantly CD14^+^ macrophages, CD3^+^ T cells, and to a lesser extent CD56^+^ natural killer (NK) cells, and CD19^+^ B cells.

## 2. Materials and Methods

### 2.1. Patient Material

Meningioma tissue was collected at Rigshospitalet at the Department of Neurosurgery (Copenhagen, Denmark) during tumor resection surgery from consenting patients. The collected meningioma tissues were stored on ice at 4 °C in phosphate buffer saline (PBS) for less than 24 h and either digested the same day or the day after resection. For extended storage of 72 h, tissues were either initially stored in PBS overnight before being transferred to Gibco™ Hibernate™-A Medium (Thermo Fisher Scientific, Waltham, MA, USA) or stored immediately in Gibco™ Hibernate™-A Medium for long-term preservation. Ethical approval was granted by the Danish Science Ethics Committee (De Videnskabsetiske Komiteer for Region Hovedstaden) with reference number H-19089882. The handling and storage of patient data and tissue material were approved by the Danish Knowledge Center for Data Reviews (j.nr: VD-P-2020-664). This study includes meningioma tissues from 28 patients with WHO 1 meningioma, 6 patients with WHO 2 meningioma, and 1 patient with WHO 3 meningioma (Table 1).

### 2.2. Digestion of Meningioma into a Viable Single-Cell Suspension

**Figure 1:**
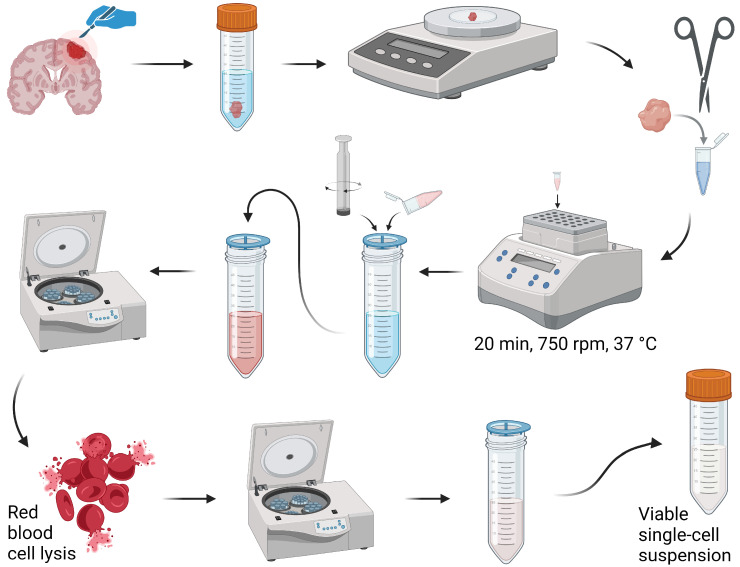
An overview of the optimized protocol for digestion of meningioma for viable single-cell suspensions comprising immune cells. The illustration was created using BioRender.

For the digestion of meningioma into a single-cell suspension (Figure 1), meningioma tissue was placed in a 1.5 mL tube containing the reagents from a Human Whole Skin Dissociation Kit (Miltenyi Biotec, Bergisch Gladbach, Germany) and 40 U/mL DNase I (Stemcell Technologies, Vancouver, BC, Canada) and cut into 1–2 mm pieces using a pair of scissors. For every 0.5 g of tissue, 300 µL of total reagents (Enzyme D, Enzyme A, and Buffer L) was used. Enzyme P was excluded following the manufacturer’s protocol, which states not to use if conserving the epitopes of the processed cells is important. The tubes were incubated at 37 °C for 20 min in a thermal shaker set at 750 rpm. The samples were forced through a 70 µm filter using the rubbery end of a plunger. The cell suspension was passed through another 70 µm filter and centrifuged at 350× *g* for 8 min. To lyse red blood cells, the cells were incubated in sterile Milli-Q water for 7 sec and washed using BSA buffer (PBS supplemented with 2% BSA buffer (Merck KGaA, Darmstadt, Germany) and 2 mM UltraPure™ EDTA (Invitrogen by Life Technology, Carlsbad, CA, USA)). The cells were resuspended in BSA buffer and placed on ice until use. Before use, the cell suspension was passed through another 70 µm filter.

### 2.3. Assessment of Immune Cell Populations Using Flow Cytometry

To label the cells for flow cytometry analyses, the cells were stained using LIVE/DEAD™ Fixable Viability Dye Zombie Green (BioLegend, San Diego, CA, USA), a LIVE/DEAD™ Fixable Green Dead Cell Stain Kit for 488 nm excitation, Fixable Viability Dye eFluor™ 780, or a LIVE/DEAD™ Fixable Near-IR Dead Cell Stain Kit (Invitrogen, Waltham, MA, USA) at 1:1000 in PBS at 4 °C for 15 min. Due to differences in characteristics of the acquired cells across patient samples, the viability of the cells were assessed without the debris fraction. For surface labeling, the cells were incubated with an antibody mixture in FACS buffer (PBS supplemented with 0.5% BSA and 2 mM EDTA) for 20 min at 4 °C, washed using FACS buffer, and fixed in CytoFix (BD Biosciences, Franklin Lakes, NJ, USA) for 20 min at RT. The cells were resuspended in FACS buffer, stored at 4 °C, and acquired within 2 weeks on a BD LSRFortessa™ X-20 Cell Analyzer (BD Biosciences) at the Core Facility for Flow Cytometry and Single Cell Analysis (University of Copenhagen, Copenhagen, Denmark). The samples were analyzed using FlowJo™ v10.8.0 Software for Windows (BD Life Sciences, Franklin Lakes, NJ, USA).

### 2.4. Antibodies for Flow Cytometry Analyses

The following antibodies were used for the labeling of cells: CD3 PerCPCy5.5 (UCHT1), CD3 PECy7 (UCHT1), CD4 BUV395 (SK3), CD4 APCCy7 (RPA-T4), CD11c PECy7 (BLY6), CD11c BV510 (B-ly6), CD19 Alexa Fluor-488 (HIB19), CD45 BV605 (HI30), CD45 BV510 (HI30), CD45RO APC-H7 (UCHL1), CD64 PerCPCy5.5 (10.1), HLA-DR APC (G46-6), TIM-3 BV711 (7D3) (BD Biosciences), CD8 BV510 (SK1), CD8 PECy7 (SK1), CD45 PECy7 (HI30), CD56 BV421 (HCD56), CD56 APC (5.1H11), CD83 APCCy7 (HB15e), TCRa/b BV421 (IP26) (BioLegend), CD4 APC (RPA-TA), CD19 PECy7 (HIB19) (Invitrogen), CD3 BV421 (REA613), CD3 APC (BW264/56), CD8 BV421 (REA734), CD11c BV421 (REA618), CD11c APC-Vio770 (REA618), CD14 APC-Vio770 (TÜK4), CD14 PE (TÜK), CD45 APC-Vio770 (REA747), HLA-DR PE (REA805) (Miltenyi Biotec), and CD45RA PerCPCy5.5 (HI100) (ProSci Incorporated, Poway, CA, USA).

### 2.5. Statistical Analyses

Statistical analyses were performed using Prism version 9.2 (GraphPad Software, La Jolla, CA, USA). Data are presented as individual values, mean or mean with standard error of the mean (SEM). A *t*-test was used to compare the mean of two groups, and one-way ANOVA with post-hoc testing was used to compare multiple groups. Statistical significances are presented as ns = non-significant, * = *p* < 0.05, ** = *p* < 0.01, *** = *p* < 0.001, and **** = *p* < 0.0001.

## 3. Results

### 3.1. Digestion of Meningioma Tissue

**Figure 2:**
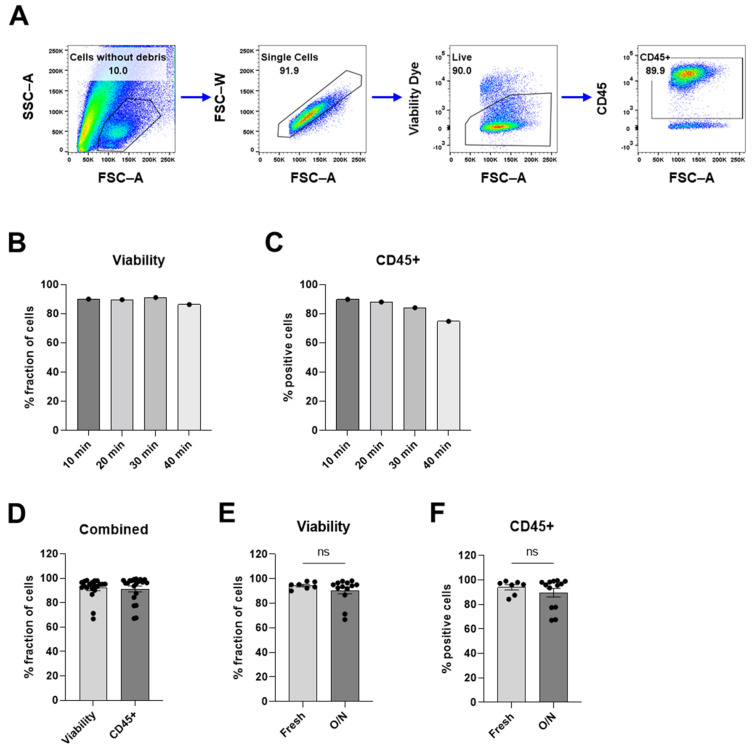
Assessment of cell suspension after digesting meningioma using flow cytometry. (**A**) Gating strategy for the samples from flow cytometry analyses. (**B**) Viability of single cells without debris clouds and (**C**) proportion of CD45^+^ immune cells after meningioma tumors were incubated in digestion reagents for 10, 20, 30, and 40 min. See Supplementary Figure S1 for more data. (**D**) The viability of single cell without debris clouds, and the proportion of CD45^+^ immune cells of digested meningioma samples. (**E**,**F**) Samples digested the same day (fresh) compared to samples digested the next day (O/N) assessed (**E**) via the viability of single cells and (**F**) via the proportion of CD45^+^ immune cells. Data is presented as individual values ((**B**,**C**) with *n* = 1 from 1 experiment) or as mean with SEM ((**D**) with *n* = 20 from 15 experiments, and (**E**,**F**) with *n* = 7 from 7 experiments) for Fresh and *n* = 13 with 9 experiments for O/N. An unpaired *t*-test was used for statistical comparisons. Statistical significances are presented as ns = non-significant.

A protocol for the digestion of meningioma tissue into a single-cell suspension with viable immune cells was optimized for the purpose of investigating tumor-infiltrating immune cells in meningioma. The texture of meningioma tissue varies between patients and ranges from very soft to stiffer fibrotic or even calcified, which makes it impossible to optimize a digestion protocol for all textures. Since this optimized protocol was suitable for soft to medium stiff tissues (most common), calcified tumors were not included in this study. Briefly, the meningioma tissue was digested using a modified protocol of a commercial kit intended for whole skin. The cells of the cell suspensions were assessed using flow cytometry to evaluate the feasibility of the optimized protocol. Debris, doublets, and dead cells were excluded from all flow cytometry analyses (Figure 2A).

For the optimization of the digestion time, the samples were incubated in dissociation reagents for 10, 20, 30, and 40 min (Figure 2B,C). To assess the optimization of the protocol, the cell suspension was analyzed for the viability of the cell suspension and the proportion of immune cells. Our results showed that a longer digestion time led to decreased viability and lower proportions of immune cells (Figure 2B,C). Incubation times up to 90 min were tested with similar results (Supplementary Figure S1). After a digestion time of 20 min, the analyzed single-cell suspension was gated to exclude debris (Figure 2A); this fraction of the cell suspension had a high viability (91.9%), and the viable cells were predominantly CD45^+^ immune cells (91.2%) (Figure 2D). Tumors that were stored on ice in PBS overnight and digested the day after resection showed similar viability and proportion of immune cells as tumors digested the same day as the resection with less than 4.5% difference; fresh vs. overnight: 94.5% vs. 90.5% for viability and 94.0% vs. 89.6% for the proportion of immune cells (Figure 2E,F). For the rest of the studies, the tumor tissue was digested for 20 min, unless the texture of the tissue was not soft enough to push through a 70 µm strainer. In that case, additional incubation was increased in 10-min increments until the tissue was soft enough to pass through a 70 µm strainer (see Table 1). The cell suspension contains a substantial quantity of debris (Figure A), which, in theory, could be reduced by additional washing steps with large washing volumes. However, the debris did not impact the analysis of the immune cells (Supplementary Figures S2 and S3). The cell suspension contains few doublets, indicating that most of the cells were present as single cells (Figure 2A). Our results showed that this protocol provides a single-cell suspension containing viable immune cells.

### 3.2. Myeloid Compartment

**Figure 3:**
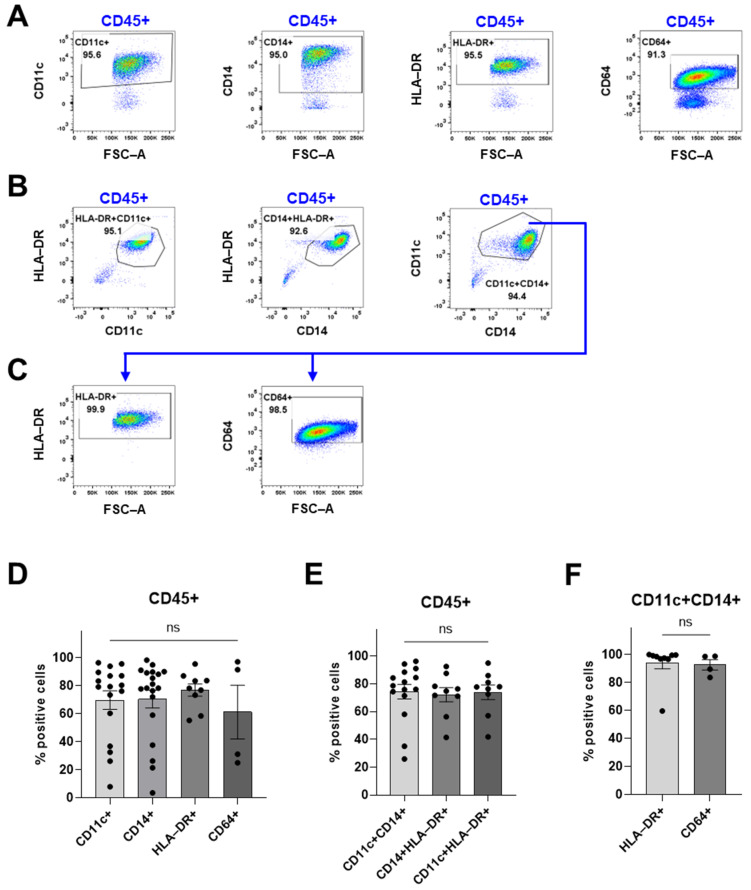
An overview of the myeloid compartment of the digested meningioma tissue using flow cytometry. Debris, doublets, and dead cells were excluded before the CD45^+^ gate (as shown in Figure 2A). (**A**) The gating strategy for Figure 3D. (**B**) The gating strategy for Figure 3E. (**C**) The gating strategy for Figure 3F. (**D**) The proportion of CD11c^+^ cells, CD14^+^ cells, and CD64^+^ cells of viable CD45^+^ immune cells. (**E**) Proportions of CD11c^+^CD14^+^, CD14^+^HLA-DR^+^, and CD11c^+^HLA-DR^+^ cells. (**F**) HLA-DR^+^ and CD64^+^ cells of CD11c^+^CD14^+^ cells. Data is presented as mean with SEM ((**D**) with *n* = 17 from 10 experiments for CD11c, *n* = 19 from 12 experiments for CD14, *n* = 9 from 6 experiments for HLA-DR, and *n* = 4 from 2 experiments for CD64; (**E**) with *n* = 15 from 9 experiments for CD11c^+^CD14^+^, *n* = 9 from 6 experiments for CD14^+^HLA-DR^+^, and *n* = 9 from 6 experiments; and (**F**) with *n* = 9 from 6 experiments for HLA-DR and *n* = 4 from 2 experiments. Either a *t*-test or one-way ANOVA with post-hoc testing was used for statistical comparisons. Statistical significances are presented as ns = non-significant.

To confirm that this protocol produces a cell suspension that is suitable for further studies of immune cells, we performed flow cytometry analyses to identify the tumor-infiltrating immune cell populations. To investigate the myeloid compartment in the tumor microenvironment, the processed cells were labeled with antibodies against CD14, CD11c, HLA-DR, and CD64 (Figure 3). The flow cytometry analyses revealed that the immune cells mainly consist of CD11c^+^, CD14^+^, HLA-DR^+^, and CD64^+^ cells (Figure 3D). To further characterize the CD14^+^ cells, different combinations of markers were explored. The proportion of CD14^+^CD11c^+^ was also CD11c^+^HLA-DR^+^ and CD14^+^HLA-DR^+^ (Figure 3E), which suggests that these cells belong to the same cell population. Moreover, the majority of the CD14^+^CD11c^+^ cells were HLA-DR^+^ and CD64^+^ as well (Figure 3F). CD64 is typically not expressed on dendritic cells but on monocytes and macrophages [23] and is considered an activation marker on macrophages [24]. Since these cells were also CD14 high and CD11c intermediate as seen in the gating strategy (Figure 3A–C), this suggests that the cells are macrophages, consistent with previous literature demonstrating the presence of macrophages in meningiomas [25,26].

**Figure 4:**
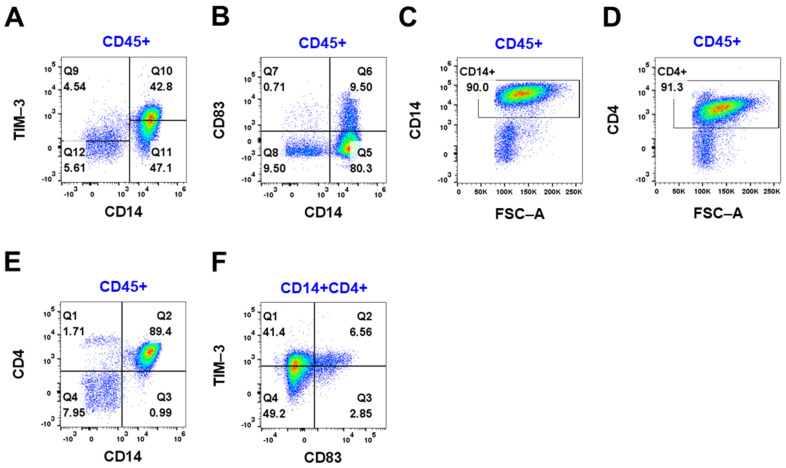
The expression of TIM-3 and CD83 in CD45^+^ immune cells was assessed using flow cytometry. (**A**) CD14/TIM-3 of viable CD45^+^ cells. (**B**) CD14/CD83 of viable CD45^+^ immune cells. (**C**) CD14 of viable CD45^+^ cells. (**D**) CD4 of viable CD45^+^ cells. (**E**) CD14/CD4 of viable CD45^+^ cells. (**F**) CD83/TIM-3 of CD14^+^CD4^+^ cells. Representative data for 2 patients with WHO grade 1 meningioma from 2 experiments.

Further investigations of the immune cells and macrophages in two meningiomas revealed expression of the immune checkpoint inhibitor TIM-3 and CD83, an activation marker of M1 macrophages (Figure 4 and Supplementary Figure S4). Half of the immune cells were TIM-3^+^ cells (47.3%) (Figure 4A), whereas 10.2% of the immune cells were CD83^+^ (Figure 4B). All CD14-positive cells were double-positive for CD4 (Figure 4C–E). Of these cells, 41.4% were TIM-3^+^CD83^-^ cells and 6.56% were positive for both TIM-3 and CD83 (Figure 4F). The majority (69.7%) of the CD83^+^ cells were double-positive for TIM-3. This suggests that the TIM-3^+^ macrophages may have an immunosuppressive role.

### 3.3. Lymphoid Compartment

**Figure 5:**
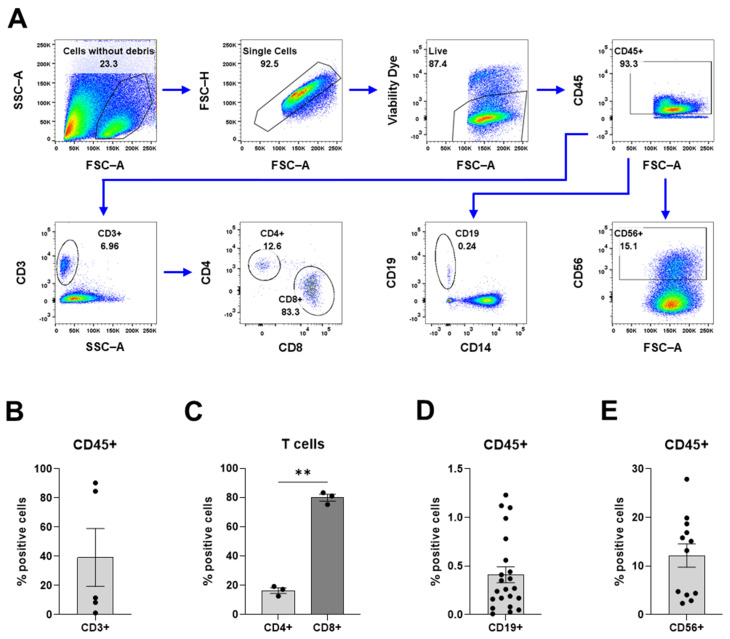
Overview of the lymphoid compartment. (**A**) The gating strategy for the flow cytometric analyses. (**B**) T cell proportion of CD45^+^ immune cells. (**C**) The proportion of CD4^+^ helper T cells and CD8^+^ cytotoxic T cells. (**D**) The proportion of CD19^+^ B cells. (**E**) The proportion of CD56^+^ NK cells. Data is presented as mean with SEM ((**B**) with *n* = 5 from 4 experiments, (**C**) with *n* = 3 from 3 experiments, (**D**) with *n* = 22 from 14 experiments, and (**E**) with *n* = 12 from 7 experiments). A *t*-test was used for statistical comparison. Statistical significances are presented as ** = *p* < 0.01.

To investigate the lymphoid compartment within the tumor, the processed cells were labeled with antibodies against CD3, CD4, CD8, CD19, and CD56 (Figure 5). Our findings revealed a large variation in the proportion of CD3^+^ T cells between the tumors; CD3^+^ cells accounted for 1.2–90.4% of all immune cells (Figure 5B). The proportion of CD3^+^ T cells correlated inversely with the proportion of macrophages (Supplementary Figure S5). The majority of the T cells were identified as CD8^+^ cytotoxic T cells (79.8%), while CD4^+^ T helper cells comprised 16.3% of the T cells (Figure 5C). The variation in the proportions of CD3^+^ T cells suggests that there is a large variability between the meningioma samples and/or within the tumors. CD19^+^ B cells (>1.5%) and CD56^+^ NK cells (12.2%) were present to a lesser extent (Figure 5D,E). Interestingly, the CD3^+^ T cells, B cells, and NK cells appeared as dichotomized clusters of either high or low proportions (Figure 5B,D,E).

### 3.4. Characterization of the Tumor-Infiltrating Immune Cells

**Figure 6:**
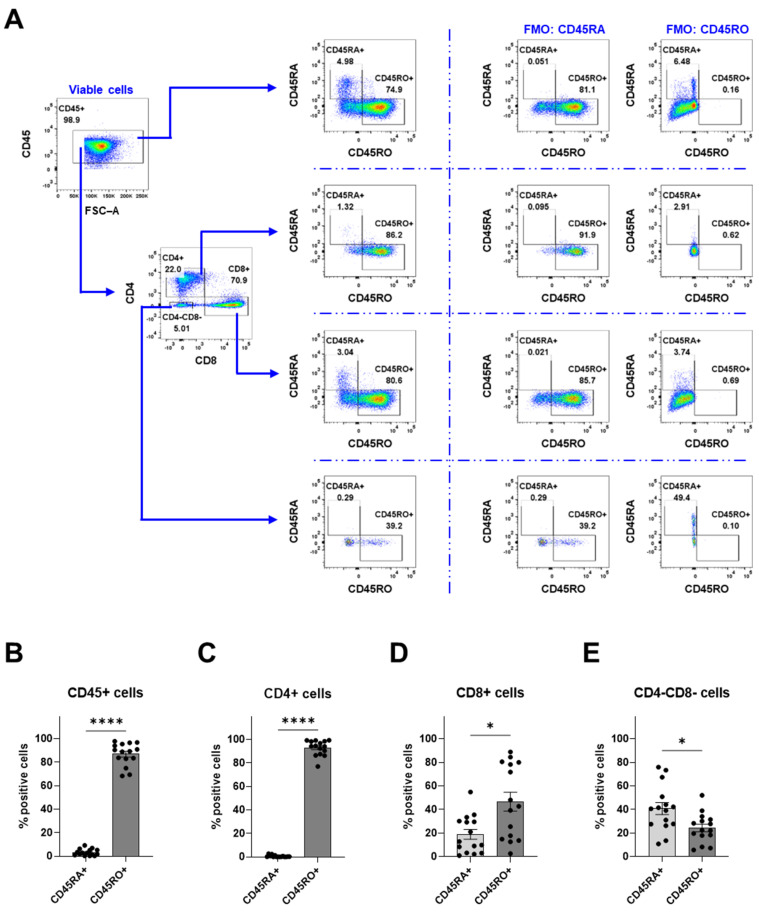
Assessment of CD45RA and CD45RO expression on immune cells within meningioma. (**A**) The gating strategy for the flow cytometry analyses. The proportion of CD45RA and CD45RO of the following populations: (**B**) CD45^+^ cells, (**C**) CD4^+^ cells, (**D**) CD8^+^ cells, and (**E**) CD4^−^CD8^−^ cells. Data is presented as mean with SEM ((**B**–**E**) with *n* = 15 from 13 experiments), and a *t*-test was used for statistical comparisons. Statistical significances are presented as * = *p* < 0.05 and **** = *p* < 0.0001.

To further characterize the tumor-infiltrating immune cells, the cells were labeled with CD45RA and CD45RO (Figure 6). The majority of the immune cells within the tumor were CD45RO^+^ immune cells (86.9%), while CD45RA^+^ cells accounted for 3.2% (Figure 6B). From the CD4 compartment that consists of both CD4^+^ T helper cells and CD4^+^ macrophages, most of the cells were CD45RO^+^ cells (Figure 6C). However, among the CD8^+^ cytotoxic T cells, CD45RO^+^ cells accounted for 46.8% and CD45RA^+^ cells accounted for 18.9% (Figure 6D). Interestingly, the CD4^-^CD8^-^ population had a lower ratio of CD45RO^+^ cells (24.3%) to CD45RA^+^ cells (40.9%) (Figure 6E). This finding shows that the majority of the immune cells expressed CD45RO.

### 3.5. Correlations

Although this study has a small cohort of patients with meningioma, we found some correlations. The parameters with clinical correlations were compiled into a table with some additional information on the samples (Table 2). Correlations were found between gender and the immune cell populations. Out of the viable cells, female patients were more likely to have a proportion of CD45^+^ cells that was lower than 90% (8 women and 1 man) whereas both women and men were found among proportions above 90% (11 women and 9 men). The proportions of CD14^+^ cells above 88% were higher for women (7 women), whereas both genders were found in proportions below 88% (5 women and 7 men). The patients with high proportions of CD14^+^ were in the group with high proportions of immune cells. The group with high proportions of CD45^+^ cells, however, did not all have high proportions of CD14^+^ cells. In the lymphoid compartment, women were more likely to have lower proportions of CD19^+^ B cells and CD56^+^ NK cells. From a total of 16 women and 6 men, there were 9 women with proportions below 0.2% for B cells. For NK cells, there were 4 women and 1 man with proportions less than 5%. In the higher proportions of NK cells above 13%, there were 4 women and 4 men. Although CD3^+^ T cells formed dichotomized clusters, no clinical correlations were observed. The identified correlations may provide insight into why women are more predisposed to meningioma.

To summarize the findings, the flow cytometry analyses revealed that the optimized protocol provides a single-cell suspension with viable immune cells that mainly consisted of CD14^+^ macrophages, CD3^+^ T cells, and, to a lesser extent, CD19^+^ B cells and CD56^+^ NK cells (Supplementary Figure S5). The CD14^+^ cells were positive for CD11c, HLA-DR, and CD4. Half of the CD14^+^ cells expressed TIM-3, and a small proportion of these cells expressed CD83. Although there was a large variation in the proportion of CD3^+^ T cells between the tumors, the majority of the CD3^+^ T cells were CD8^+^ cytotoxic T cells. The tumor-infiltrating immune cells were mainly CD45RO^+^ cells. Women were more likely to have a lower proportion of immune cells, and the female patients with a high proportion of immune cells had a higher proportion of macrophages. Women were also more likely than men to have lower proportions of B cells and NK cells.

## 4. Discussion

An optimized protocol for digesting meningioma tissue into a single-cell suspension of viable tumor-infiltrating immune cells is presented herein along with the characterization of the infiltrated immune cell populations.

Meningioma tissues were successfully digested into a single-cell suspension with viable immune cells. Due to the variations in size and granularity of the acquired cells across patients, the voltages for FSC, SSC, and threshold were adjusted accordingly. Furthermore, the debris fraction was also influenced by washing volumes and, thus, the number of times red blood cells were lysed. Additionally, each piece of debris would be counted as a separate event, even if the debris originated from a single cell, which would further skew the percentages. Therefore, the viability of the total cell suspension could not be accurately assessed with debris included. Consequently, we assessed the viability of the cells without the debris in this study. Flow cytometry analyses revealed that immune cells made up a fraction of 91.2% of the total viable cells with various immune cell populations. The largest population of immune cells was tumor-associated macrophages (70.5%) that co-expressed CD14, CD4, CD11c, CD64, and HLA-DR. The high-affinity IgG receptor CD64 can be used as a marker for identifying activated macrophages and is associated with inflammatory responses [24,27]. CD64 is typically perceived as an M1 marker; however, M2 macrophages also express this receptor at lower expression levels. We did not attempt to describe the M1/M2 activation status of the associated macrophage population; however, previous studies using immunohistochemistry have shown that the majority of tumor-associated macrophages are of the M2 phenotype [25]. Using immunofluorescence staining, it has previously been shown that tumor-associated macrophages were the predominant immune cell type in meningioma [25,26]. The largest population of tumor-infiltrating lymphocytes were T cells (39.2%), predominantly CD8^+^ T cytotoxic T lymphocytes (79.8%). Other cell populations found to a much lesser extent were B cells (0.42%) and NK cells (12.2%). These findings are in line with previous studies of meningioma cell populations [25,28,29,30].

Although only two patients with WHO 1 meningothelial meningioma were investigated for TIM-3 expression, half of the macrophages from these patients interestingly expressed TIM-3, an immune checkpoint receptor that is involved in the regulation of immune tolerance [31]. In a retrospective cohort study, high TIM-3 expression was associated with a poorer prognosis for patients with non-small cell lung cancer [32]. TIM-3 has been suggested to have a prognostic value in solid tumors as high expression of TIM-3 was associated with poor overall survival [33]. A smaller proportion of these TIM-3^+^ cells were also CD83^+^. CD83 expression on macrophages has been reported to limit inflammation in a wound-healing model in mice [34]. This suggests that both TIM3^+^ and TIM3^+^CD83^+^ macrophages in meningioma may have an immunosuppressive role. CD45RO is typically not expressed on macrophages; however, CD45RO^+^ macrophages have been observed in HIV-1 encephalitis, indicating that CD45RO plays an inflammatory role in macrophages under certain pathological conditions or in inflammatory environments [35]. As most of the tumor-infiltrating immune cells were CD45RO^+^ cells, and the majority of the CD4+ cells are macrophages, this would imply that the macrophages within the tumor microenvironment are in an inflammatory or activated state.

Tumor-infiltrating immune cells, including the tumor-infiltrating lymphocytes, play a key role in tumor development and immune evasion. T cells and B cells have previously been reported in meningioma using immunohistochemistry [30], and the proportion of CD8^+^ T cells was higher than the proportion of CD4^+^ T cells [30,36]. CD8^+^ T cells play a crucial role in anti-tumor immunity [37] and eliminate tumors through several mechanisms such as inducing apoptotic cell death via perforin-granzyme or Fas-FasL interaction and by secreting cytokines such as IFN-ɣ and TNF-α within the TME [38]. Tumors with a substantial presence of tumor-infiltrating lymphocytes, especially in connection to PD-1 expression, are called hot tumors, while tumors with poor infiltration are termed cold tumors [39]. How hot or cold a tumor is can be used to predict a tumor’s ability to respond to immune checkpoint inhibitors [39]. For instance, low CD8^+^ T cell numbers were linked to a high recurrence rate [40], while higher numbers of CD8^+^ T cells were associated with improved progression-free survival [41]. Interestingly, 40.8% of CD8+ T cells in meningioma was CD45RA^+^. CD45RA expression on T cells is normally considered a marker for naïve T cells which are CD45RA^+^CCR7^+^CD62L^+^ cells [42]. However, recently differentiated T cells can regain CD45RA expression [43] and are known as T effector memory re-expressing CD45RA (T_EMRA_) cells and defined as the most terminally differentiated subset [44]. These CD45RA^+^ effector memory cells are also CCR7^-^ and can recognize antigens in the tissues for rapid effector responses [45]. They have been found in glioblastoma and in non-small cell lung cancer [44,45,46]. NK cells have also been previously identified in meningioma using conventional mechanical disaggregation [47]. It is an important lymphocyte subpopulation capable of directly killing cancer cells without prior sensitization, making them a promising option for immunotherapy against tumors [48,49]. Intriguingly, the lymphocyte populations appeared as high- or low-proportion dichotomized clusters, and the proportion of T cells correlated inversely with the proportion of macrophages; however, no correlation to grade, subtype, location, or gender was found.

### 4.1. Development of the Tissue Digestion Protocol

Most of the early work was performed using a conventional mechanical disaggregation procedure known as fine needle aspiration [50]. Since then, several protocols for digesting meningioma into single-cell suspensions have been reported [21,47]. For single-cell RNA sequencing, Wang et al. reported on two protocols for the digestion of meningioma, one overnight protocol containing Collagenase D, among other reagents, and a commercial kit intended for single-cell tumor dissociation [21]. The commercial tumor dissociation kit is for purifying tumor cells from tissues, which was reflected in our tests as the tumor dissociation kit had higher proportions of CD45^-^ cells and lower proportions of CD14^+^ cells compared to the optimized protocol (Supplementary Figure S6). Surprisingly, the CD8+ populations were missing in the cell suspension from the tumor dissociation kit, which is therefore not optimal for studying the immune cell composition of meningioma tissues. Another advantage of the protocol proposed herein is the shorter incubation time for digestion: 20 min. This minimizes the time the cells are exposed to suboptimal conditions and removed from the tumor microenvironment. Reducing the overall processing time allows for more time-intensive analysis protocols that may follow the digestion.

During optimization, two digestion protocols were tested, namely Collagenase D (Supplementary Figure S7) and the commercial kit developed for generating single-cell suspensions from human skin biopsies. Given that skin is composed of relatively stiff tissue, we hypothesized that this commercial kit would be effective on meningioma tissues with medium stiffness. Meningioma tissue digested using Collagenase D resulted in a cell suspension with a reduced yield of immune cells compared to the skin digestion kit (Supplementary Figure S7). Due to the difficulty of obtaining patient material and large tissue samples, we decided to use the commercial digestion kit for optimizations. This kit contains enzymes that degrade both extracellular adhesion proteins and the extracellular matrix. Additionally, it has been shown to generate cells suitable for downstream applications, including cell cultivation [51] and single-cell RNA sequencing [52]. Different incubation times were tested to account for varying tissue textures of meningiomas, and both the viability and proportion of immune cells were assessed to shorten the length of the protocol. To mitigate cell aggregations, DNase was included during the digestion to break down extracellular DNA, while EDTA was added to the BSA buffer to decrease cell-to-cell adhesion. We found that a dissociation time longer than 20 min decreased both the viability of the cells from the tumors and the proportion of immune cells in the suspension (Supplementary Figure S1). Various viable cell populations were identified using flow cytometry, which suggests that using the protocol presented herein, different cell populations can be further isolated and used for other downstream applications as well. The protocol could potentially be useful for other types of solid intracranial tumors that share similarities with meningiomas, such as schwannomas and hemangiopericytomas.

### 4.2. Limitations

A limitation of this study, other than the number of patients, includes the sample size and sampling location due to tumor heterogeneity. Therefore, analyzing larger samples is more advantageous, as it helps minimize the impact of tumor heterogeneity, which can act as a confounding factor in the analysis. Given this, studies using techniques such as immunohistochemistry, immunofluorescence, and tissue microarrays may not provide a representative image of the entire tumor. Another limitation is the uncertainty of whether the obtained single-cell suspension accurately reflects the full repertoire of immune cell populations within the tumors, or if it primarily comprises the most resilient, stress-resistant immune cells that were easiest to extract. As the extracted viable cells were mainly immune cells, it is not possible to determine the proportion of immune cells relative to tumor cells. Additional techniques, such as immunohistochemistry and multiplex immunofluorescence, may aid in verifying the diversity of the immune cell repertoire.

### 4.3. Future Studies

Future studies will focus on further characterizing the various immune cell populations and investigating their roles in tumor immune evasion, with an initial emphasis on the tumor-infiltrating CD8^+^ T cells. Our preliminary data show that the infiltrating CD8^+^ T cells in meningioma express PD-1 and/or produce IFN-ɣ, though we have not yet confirmed whether these functions are exhibited by the same population. We are currently exploring the role of soluble factors in meningioma progression using functional-cell-based assays. In addition, we are currently setting up T cell suppression assays to investigate the suppressive capacity of the tumors. After interaction with tumor cells and other components of the tumor microenvironment, the CD8^+^ T cell compartment will be extensively assessed for checkpoint inhibitors, exhaustion markers, and cytokine profiles.

In summary, the optimized protocol presented here offers a shorter digestion time for meningiomas compared to previously reported protocols, and it produces a single-cell suspension containing viable immune cells. After removing cell debris and dead cells, this suspension is suitable for downstream analyses requiring immune cells with good viability, such as cell cultivation and transcriptomic profiling using single-cell RNA sequencing. A shorter digestion protocol also allows for faster processing of patient material, which better preserves the quality of the samples. Ultimately, identifying targetable mechanisms could potentially provide patients with new non-surgical treatment options for improved therapeutic outcomes and reduce the burden on society and the community as fewer patients will need post-treatment care and can resume activities sooner.

## 5. Conclusions

To conclude, we have optimized a protocol for digesting meningioma tissues into single-cell suspensions with viable, activated immune cells, specifically, macrophages, T cells, and to a lesser extent B cells and NK cells. This streamlined protocol provides researchers with a valuable tool for obtaining single cells suitable for downstream applications, such as cell cultivation and analysis. This enables further investigations of the complex interactions between tumor and immune cells, pinpointing the mechanisms by which tumor cells manipulate immune cells to evade the immune system.

## Figures and Tables

**Table 1:** Overview of included patients.

ID#	WHO Grade	Subtype	Size (AP × SS × CC) in mm	Hemi-sphere	Edema	Location	Recurrence	Gender	Age of Patient	Weight of Obtained Tissue (g)	Digestion Time (min)
17	1	Meningothelial	57 × 46 × 49	dxt	Yes	Convexity, frontal	No	Female	63	-	90
30	1	Meningothelial	25 × 20 × 28	sin	No	Convexity, parietal	No	Female	59	1.78	20
37	1	Meningothelial	52 × 43 × 63	sin	Yes	Falx	No	Male	74	0.92	20
38	1	Meningothelial	40 × 35 × 35	dxt	Yes	Sphenoid wing	No	Male	58	0.83	20
39	1	Fibrous	40 × 35 × 37	dxt	Yes	Falx	No	Male	79	0.80	20
41	1	Meningothelial	10 × 17 × 22	bilat	No	Olfactory	No	Female	48	0.10	15
47	1	Fibrous	32 × 29 × 25	dxt	No	Convexity, frontal	No	Female	57	0.60	20
48	1	Meningothelial	34 × 23 × 21	sin	No	Convexity, frontal	No	Male	71	0.53	20
52	1	Secretory	23 × 30 × 25	dxt	Yes	Sphenoid wing	No	Female	59	0.47	20
53	1	Secretory	29 × 28 × 18	sin	Yes	Sphenoid wing	No	Female	70	0.30	20
54	1	Meningothelial	32 × 26 × 30	sin	No	Convexity, N/A	No	Female	30	0.94	20
55	1	Mixed	20 × 23 × 27	sin	-	Sphenoid wing	No	Female	74	0.77	20
58	1	Angiomatous	46 × 30 × 40	sin	Yes	Convexity, frontal	No	Female	66	1.57	20
60	1	Meningothelial	61 × 77 × 37	bilat	No	Convexity, parasagittal	No	Male	54	0.22	20
61	2	Atypic	7 × 18 × 13	dxt	No	Falx, parietal (multiple)	Yes	Female	47	0.40	20
65	3	Anaplastic	20 × 49 × 31	sin	Yes	Parietal	Yes	Female	51	0.15	20
67	1	Meningothelial	39 × 41 × 33	bilat	Yes	Convexity, frontal	No	Male	63	0.82	20
68	1	Meningothelial	28 × 14 × 35	sin	-	Sphenoid wing	Yes	Female	60	0.31	20
71	1	Fibrous	26 × 17 × 29	dxt	No	Cerebellopontine angle	No	Female	37	0.16	20
78	2	Atypic	64 × 41 × 46	sin	Yes	Frontal	Yes	Female	84	1.20	20
79	1	Meningothelial	23 × 29 × 35	sin	No	Convexity, frontal	No	Male	65	0.81	20
80	2	Atypic	32 × 32 × 16	sin	Yes	Parasagittal frontal	No	Male	64	3.75	40
81	1	Meningothelial	22 × 12 + 24 × 14	dxt	-	Dxt orbita + frontal	No	Female	31	0.61	20
90	1	Meningothelial	19 × 21 × 18	sin	-	Posterior planum sphenoidale	No	Female	74	0.54	20
91	2	Atypic	39 × 43 × 38	bilat	Yes	Falx, frontal	No	Male	68	0.42	20
92	1	Meningothelial	41 × 31 × 34	sin	No	Parafalcine, occipital/parietal	No	Female	57	0.59	20
93	1	Fibrous	27 × 22 × 29	sin	No	Convexity, frontal	No	Female	70	0.70	20
94	1	Transitional	55 × 43 × 54	sin	No	Falx, frontopariet	No	Male	42	2.36	20
95	1	Meningothelial	27 × 22 × 30	dxt	Yes	Falx, frontal	No	Male	76	1.91	20
105	2	Atypic	40 × 56 × 47	dxt	Yes	Frontal	Yes	Male	59	3.22	20
106	1	-	25 × 22 × 12	bilat	No	Planum sphenoidale	No	Female	32	0.53	20
111	1	Meningothelial	25 × 25 × 27	sin	No	Medial sphenoid wing	No	Female	59	0.84	20
114	1	Meningothelial	24 × 20 × 20	dxt	-	Prepontine cistern	No	Female	77	0.63	20
122	1	Meningothelial	35 × 32 × 42	sin	-	Tuberculum jugulare	Yes	Female	58	0.47	20
123	2	Atypic	8 × 13 × 28	sin	No	Tuberculum jugulare/foramen magnum	No	Male	61	1.47	30

**Table 2:** Selected parameters of flow cytometry analyses for each patient where correlations were found. The data are sorted by gender and subtype. Single cells were gated without debris, viability was assessed from single cells without debris, and CD45+ cells were gated from the viable cells (Figure 2A and Figure 5A). The remaining cell populations were gated from CD45+ cells (Figure 5A). Values are highlighted in orange for high fractions and in yellow for low fractions, according to the cutoff value for correlation.

ID#	WHO Grade	Gender	Subtype	Age of Patient	Single Cells (%)	Live (%)	CD45^+^ (%)	CD14^+^ (%)	CD19^+^ (%)	CD56^+^ (%)	CD3^+^ (%)
106	1	Female	-	32	95.2	99.8	85.1	-	-	2.88	11.5
65	3	Female	Anaplastic	51	96.2	95	67	77.7	0.78	-	-
58	1	Female	Angiomatous	66	96.4	97.9	99.1	-	0.16	-	-
61	2	Female	Atypic	47	94.1	93.6	98	89.2	0.19	-	-
78	2	Female	Atypic	84	94	94.2	95.5	89.8	0.44	-	-
93	1	Female	Fibrotic	70	88.9	95	84.3	37.5	0.17	16.9	-
71	1	Female	Fibrotic	37	81.5	66.8	93.5	95	0.027	-	-
47	1	Female	Fibrotic	57	-	-	-	-	-	-	-
30	1	Female	Meningothelial	59	-	-	-	-	-	-	-
111	1	Female	Meningothelial	59	89.9	94.1	99.3	90.1	-	4.07	-
114	1	Female	Meningothelial	77	84.1	77.6	97.2	88.2	-	4.41	8.41
90	1	Female	Meningothelial	74	93.8	95.3	91.3	98.3	0.065	15.1	-
92	1	Female	Meningothelial	57	93.8	94.6	94.4	91.1	0.049	27.9	-
17	1	Female	Meningothelial	63	79.6	68.7	76.7	-	0.08	-	84.6
122	1	Female	Meningothelial	58	69.1	71.2	77.8	21.4	1.12	2.33	-
41	1	Female	Meningothelial	48	-	-	-	-	-	-	-
54	1	Female	Meningothelial	30	77.3	93.2	87.6	-	0.17	-	-
81	1	Female	Meningothelial	31	84.8	92.3	95.9	59	0.99	-	-
68	1	Female	Meningothelial	60	95.5	97.9	98.9	78.3	0.26	-	-
55	1	Female	Mixed	74	93.8	96.6	99.1	-	0.41	-	-
53	1	Female	Secretory	70	91	91.7	67.6	-	0.00819	-	-
52	1	Female	Secretory	59	90.9	92.4	77.4	-	0.27	-	-
105	2	Male	Atypic	59	96.4	98.3	97.2	-	-	13.2	1.18
91	2	Male	Atypic	68	96.3	98	73.6	26.2	1.1	19.9	-
80	2	Male	Atypic	64	92.5	87.4	76.4	76.4	0.24	-	-
123	2	Male	Atypic	61	92.7	96.6	99.4	3.56	1.23	4.76	90.4
39	1	Male	Fibrotic	79	-	-	-	-	-	-	-
60	1	Male	Meningothelial	54	91.2	90.1	98	-	0.37	-	-
37	1	Male	Meningothelial	74	-	-	-	-	-	-	-
38	1	Male	Meningothelial	58	-	-	-	-	-	-	-
67	1	Male	Meningothelial	63	94.7	86.8	95.9	72	0.56	-	-
48	1	Male	Meningothelial	71	96.9	97.4	96.2	-	-	-	-
79	1	Male	Meningothelial	65	96.3	95	96.8	81.2	0.35	-	-
95	1	Male	Meningothelial	76	89.1	95.3	97.2	85.4	-	15.8	-
94	1	Male	Transitional	42	92.1	98.2	98.3	78.3	-	18.7	-

## Data Availability

Data are unavailable due to privacy or ethical restrictions.

## Supplementary Materials

The following supporting information can be downloaded at: https://www.mdpi.com/article/10.3390/cancers16233942/s1, Figure S1. Meningioma tissue from 5 patients was digested using a modified protocol of the commercially available whole-skin digestion kit. Figure S2. Comparison of cell populations when gating with and without debris. Figure S3. Backgating of different populations after gating on single cells. Figure S4. FMO controls for Figure 4. Figure S5. Pie chart of the immune cell composition in meningioma. Figure S6. For the digestion of meningioma tissue, two commercially available kits were compared: whole skin dissociation with an optimized protocol and tumor dissociation performed according to the manufacturer’s manual. Figure S7. Comparison between the optimized digestion protocol using the commercially available kit for whole skin dissociation and another digestion protocol using 0.5 mg/mL Collagenase D.
