# Supplementary material for: Flow Cytometry Analyses of Meningioma Immune Cell Composition Using a Short, Optimized Digestion Protocol
Source: Cancers (Basel). 2024 Nov 25;16(23):3942. doi: 10.3390/cancers16233942 (PMC11640484; doi:10.3390/cancers16233942)
Supplement: Supplementary file 1 [file cancers-16-03942-s001.zip › Supplementary Figures for version11.pdf]

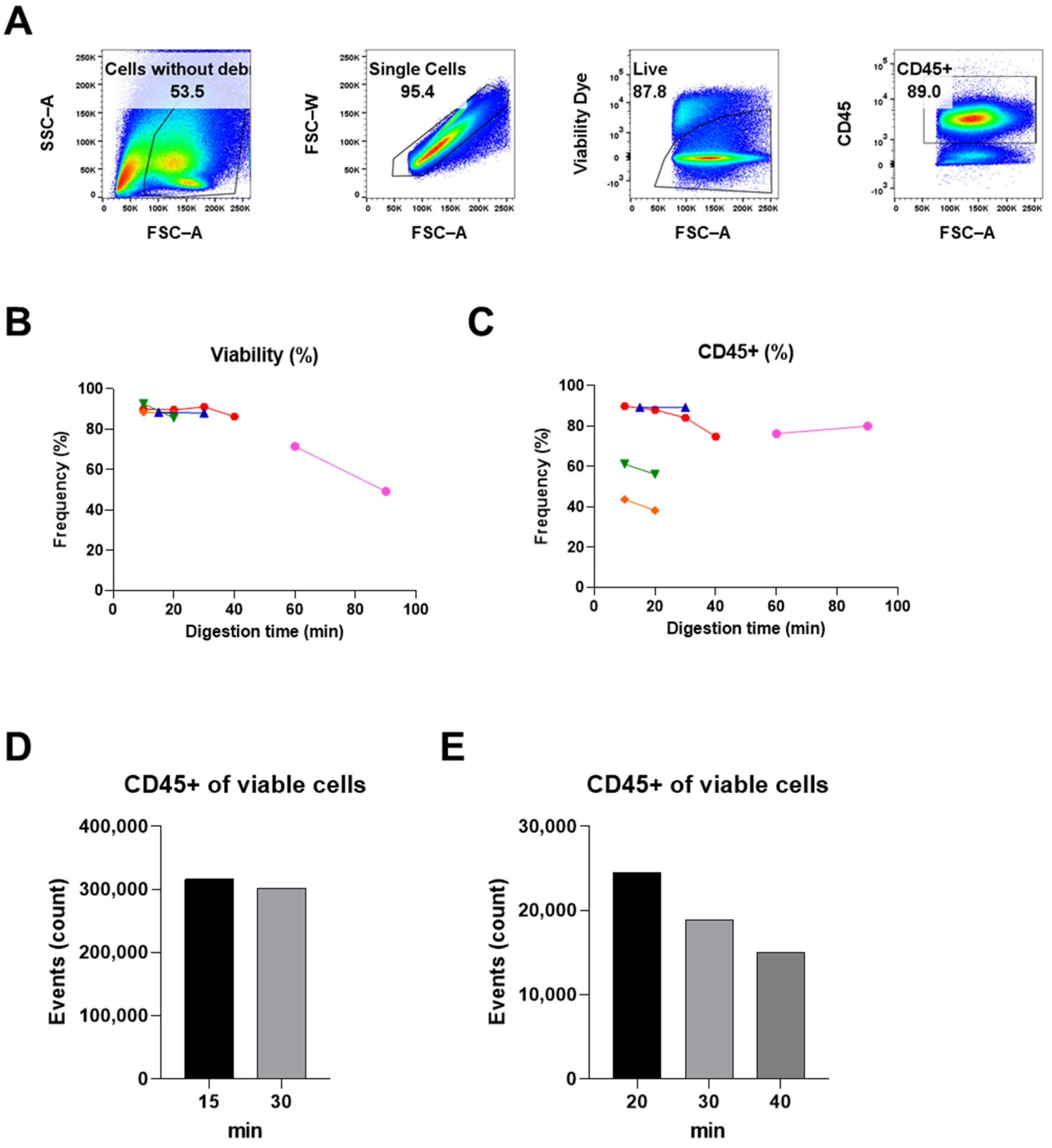

Supplementary Figure S1. Meningioma tissue from 5 patients was digested using a modified protocol of the commercially available whole-skin digestion kit. For each patient, the tissue was divided into pieces of equal weight. **(A)** The cells were gated according to the gating strategy. The optimal digestion time was assessed (color-coded for each patient) using **(B)** the viability of the cells and **(C)** the fraction of CD45+ immune cells. **(D-E)** For these two patients, the same volume was used for the acquisition on the flow cytometer. The events refer to the CD45+ immune cell population of the viable cells. Considering the viability, the fraction of CD45+ immune cells, and the event counts for the CD45+ immune cells, the optimal time for digestion was 20 min.

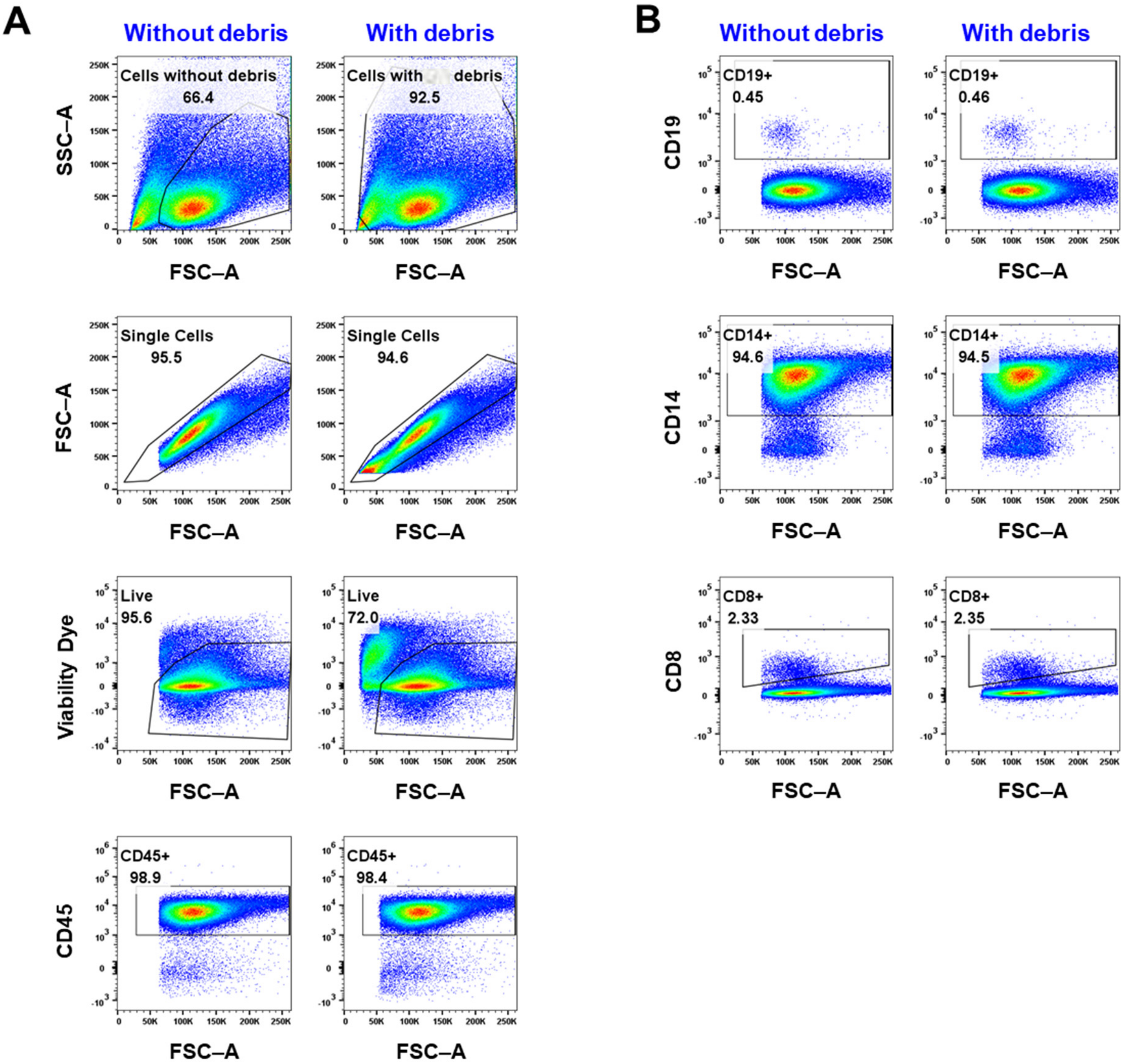

Supplementary Figure S2. Comparison of cell populations when gating with and without debris. Compared to the gating strategy without debris, the proportion of viability decreased. However, no substantial differences in proportions were found for immune cell populations when including the debris.

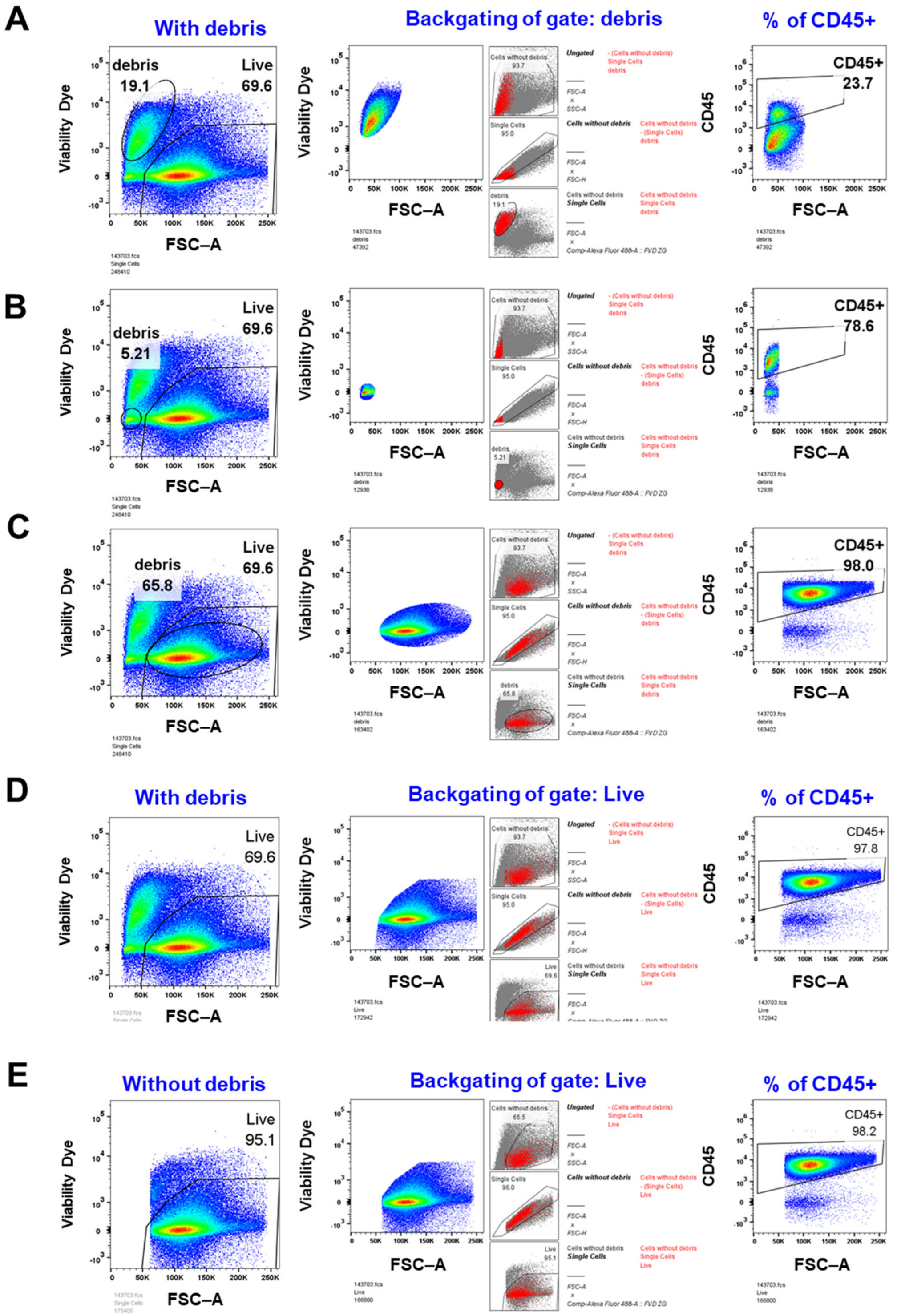

Supplementary Figure S3. Backgating of different populations after gating on single cells. **(A)** The population with high expression of the viability dye, and **(B)** the population that is low in FSC-A with low expression of viability dye belong to the cloud of debris in the first gate, *i.e.*, in the FSC-A/SSC-A gate. **(C)** The backgating of the population gated as viable cells (named debris in the plot) showed that this population is where cells are expected to be found in the FSC-A/SSC-A gate. **(D-E)** Backgating of the live-gated used in the analysis of the data in the paper, with **(D)** debris and **(E)** without debris. Both with and without including the debris, the fraction of immune cells remains the same. This shows that gating with or without the debris does not impact the fraction of immune cells.

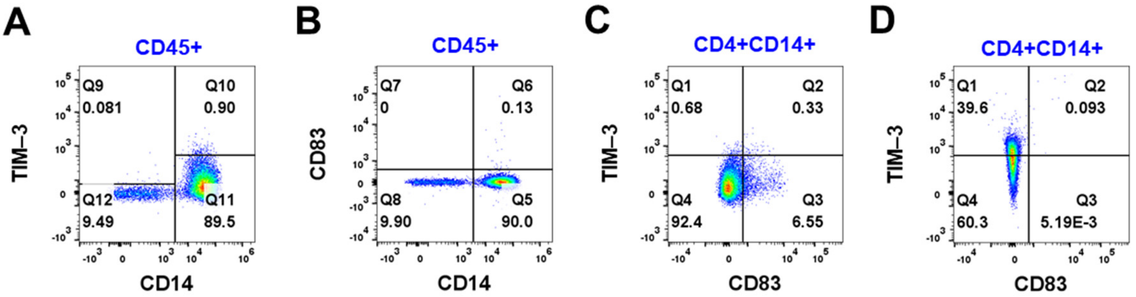

Supplementary Figure S4. FMO controls for Figure 4.

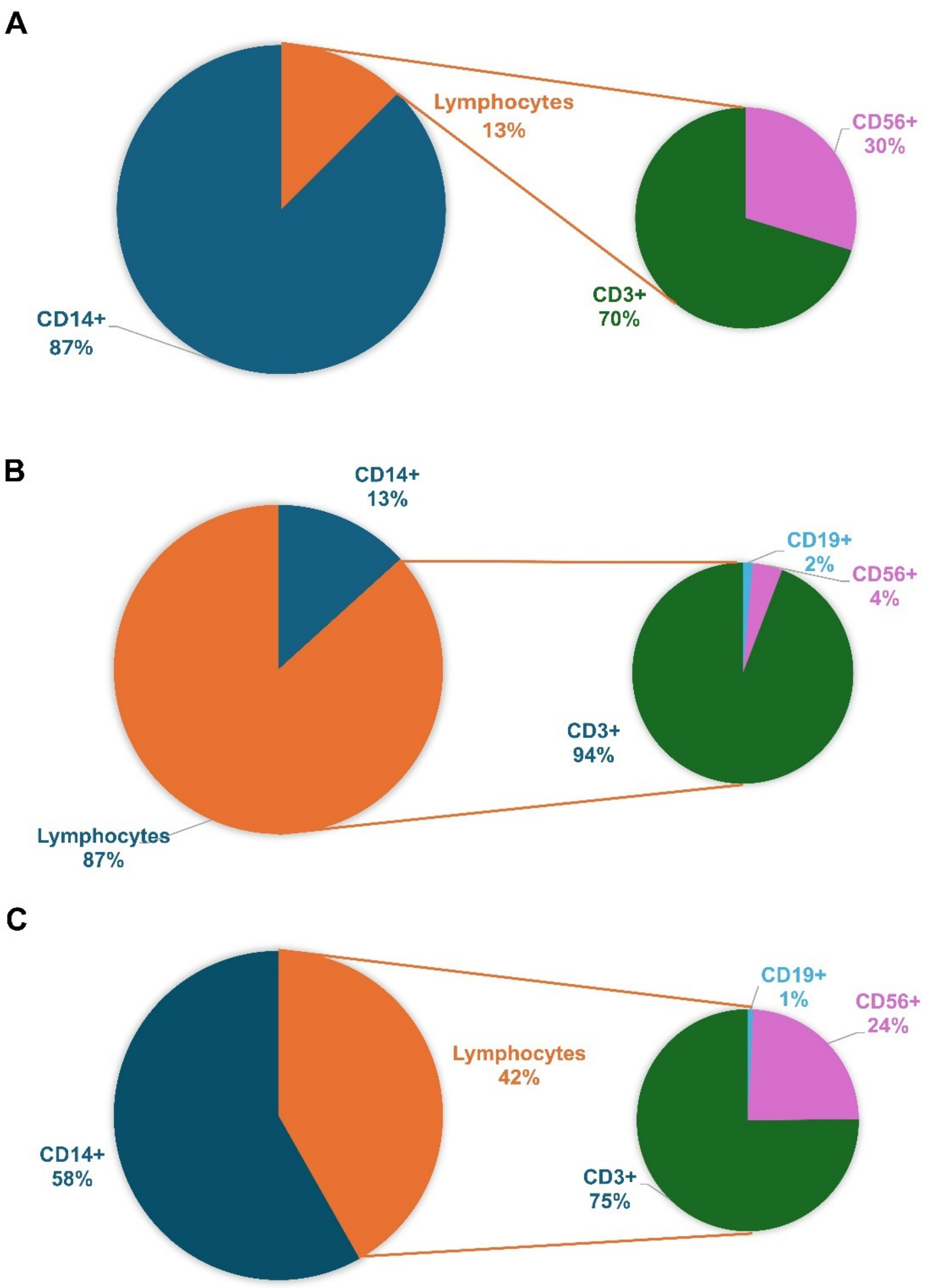

Supplementary Figure S5. Pie chart of the immune cell composition in meningioma. The proportion of T cells correlated inversely with the proportion of macrophages. **(A)** Immune cell composition of lymphocytes (CD56+ and CD3+) and CD14+ cells in patients with low proportions of CD3+ T cells. **(B)** Immune cell composition of lymphocytes (CD56+, CD3+, and CD19+) and CD14+ cells in patients with high proportions of CD3+ T cells. **(C)** Immune cell composition of all patients combined.

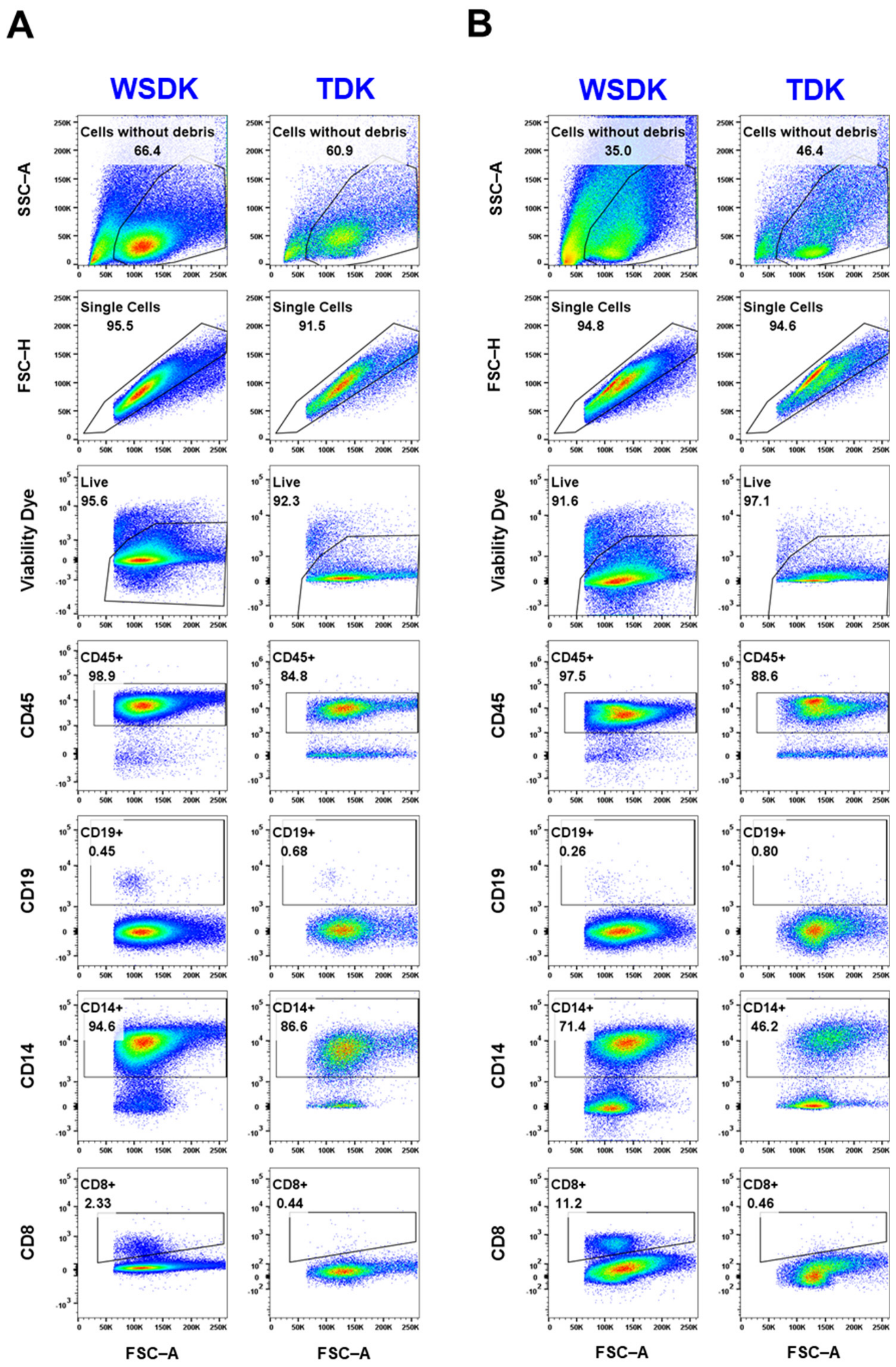

Supplementary Figure S6. For the digestion of meningioma tissue, two commercially available kits were compared: whole skin dissociation (WSDK) with an optimized protocol and tumor dissociation (TDK) performed according to the manufacturer's manual. The gating strategy and the proportions of the gated populations for both patients: **(A)** and **(B)**. Patient A had a higher proportion of viable cells when using WSDK, but Patient B had higher viability when using TDK. For both patients, the CD45+ proportion of viable cells was, however, higher when using WSDK. The proportion of CD14+ of CD45+ immune cells was higher for both patients when using the WSDK. Interestingly, CD8+ cells were not found when using the TDK. Considering this, the optimized protocol using WSDK was a better option for investigating the immune cell composition within meningiomas.

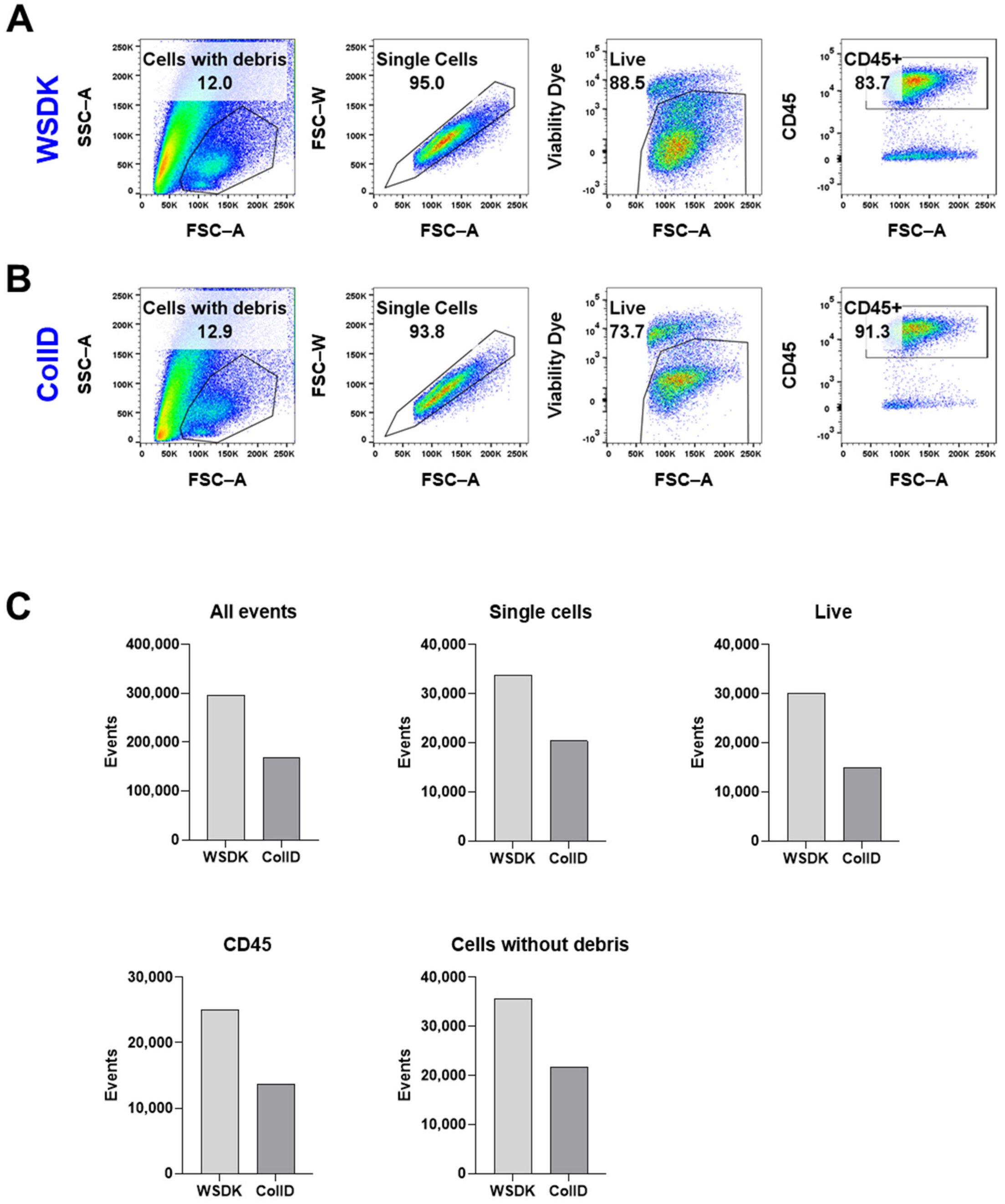

Supplementary Figure S7. Comparison between the optimized digestion protocol using the commercially available kit for whole skin dissociation (WSDK) and another digestion protocol using 0.5 mg/ml Collagenase D (CollD). The meningioma tissue was divided into two equal pieces, one for each method, and the same volume was acquired on the flow cytometer. **(A-B)** The gating strategy and the fractions of the gated populations. WSDK had better viability than CollD, but CollD had a higher fraction of CD45+ immune cells. **(C)** Event counts for WSDK were almost doubled compared to CollD. To extract as many viable immune cells as possible, the optimized protocol using WSDK performed better than the protocol using CollD.
