# Supplementary figures and images for: Flow Cytometry Analyses of Meningioma Immune Cell Composition Using a Short, Optimized Digestion Protocol
Source: Cancers (Basel). 2024 Nov 25;16(23):3942. doi: 10.3390/cancers16233942 (PMC11640484; doi:10.3390/cancers16233942)

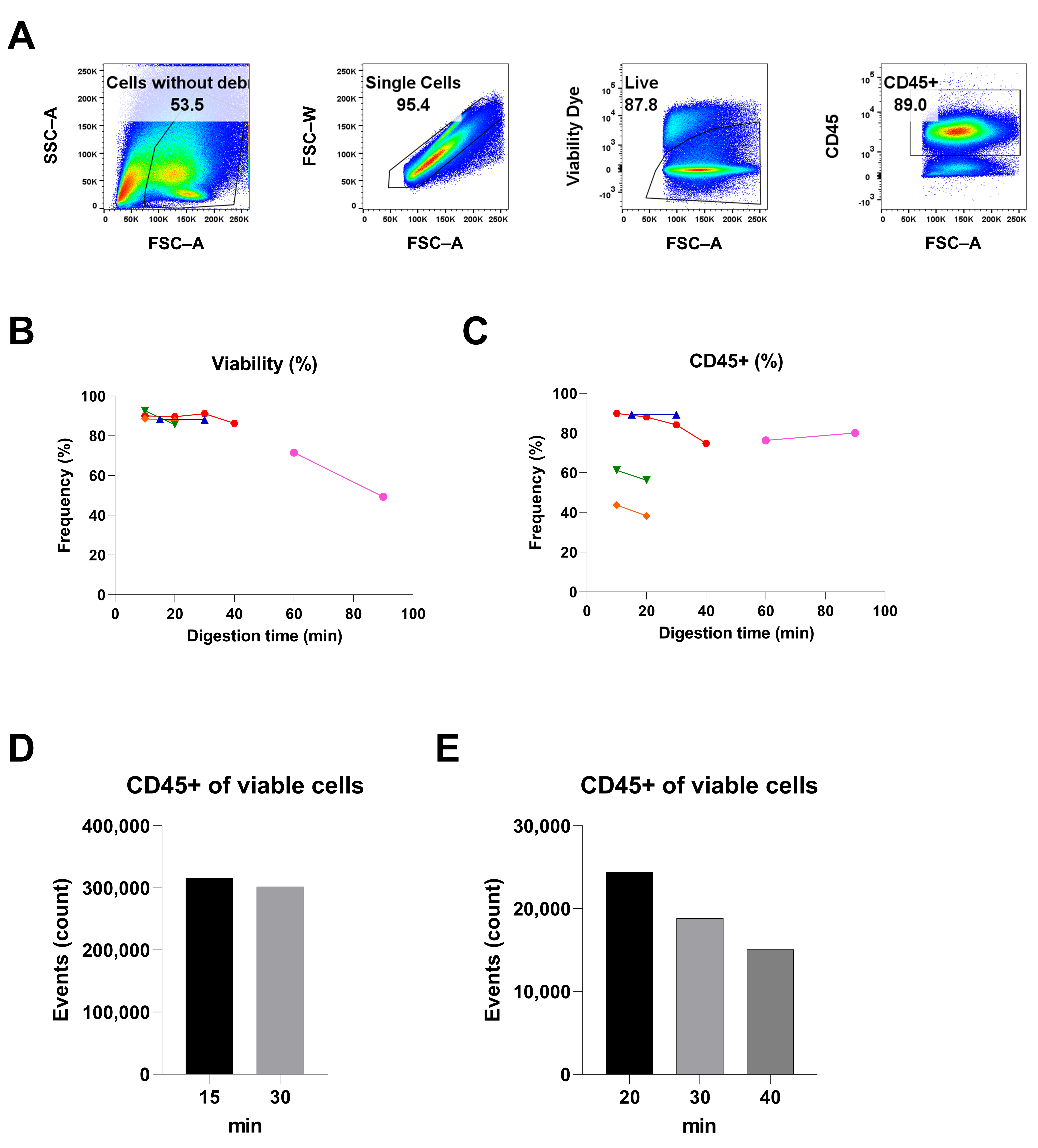

Supplement: Supplementary file 1 [file cancers-16-03942-s001.zip › Sfig1.tif]

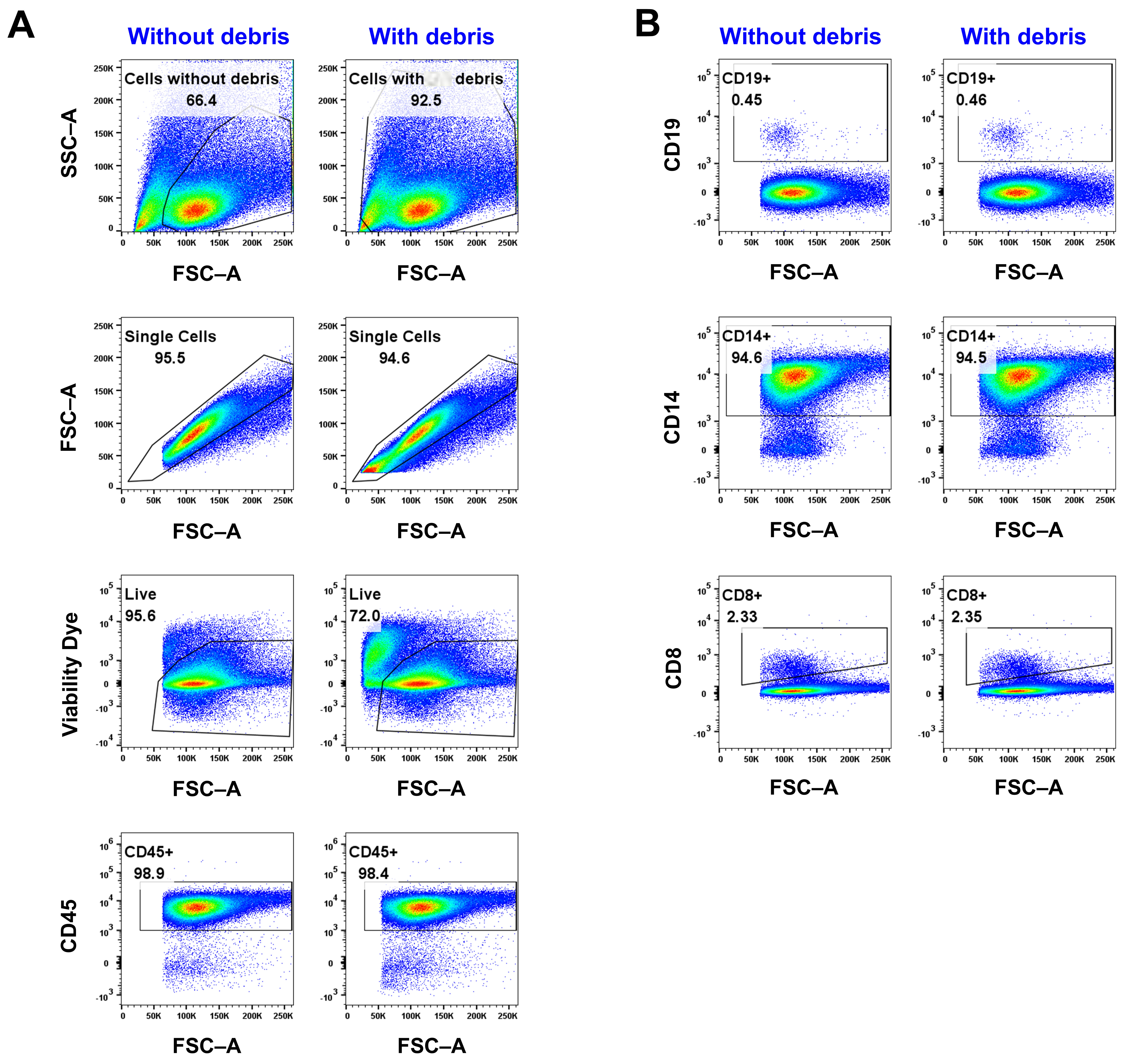

Supplement: Supplementary file 1 [file cancers-16-03942-s001.zip › SFig2.tif]

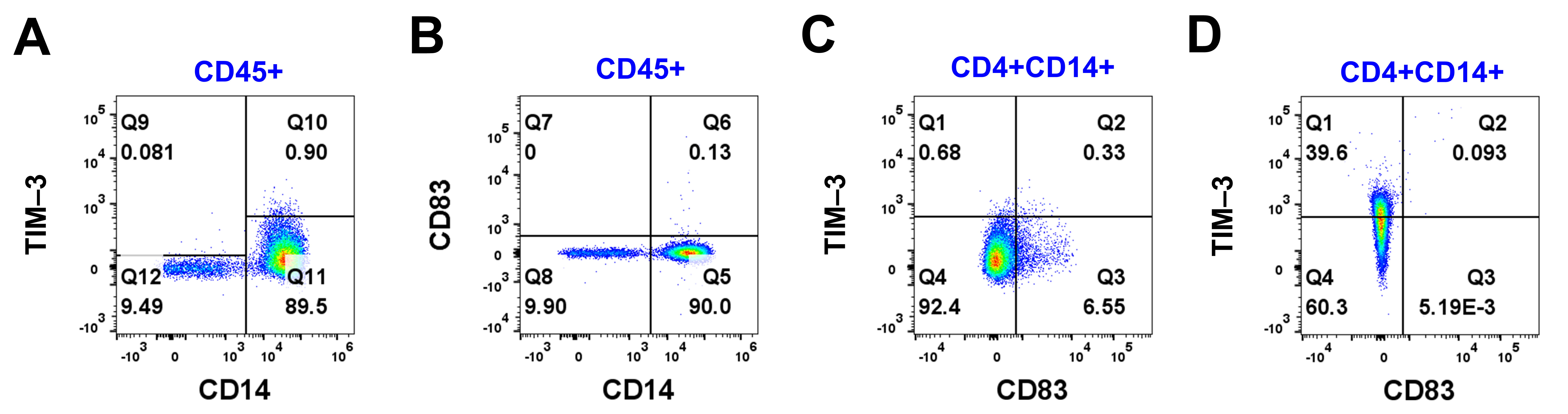

Supplement: Supplementary file 1 [file cancers-16-03942-s001.zip › SFig4.tif]

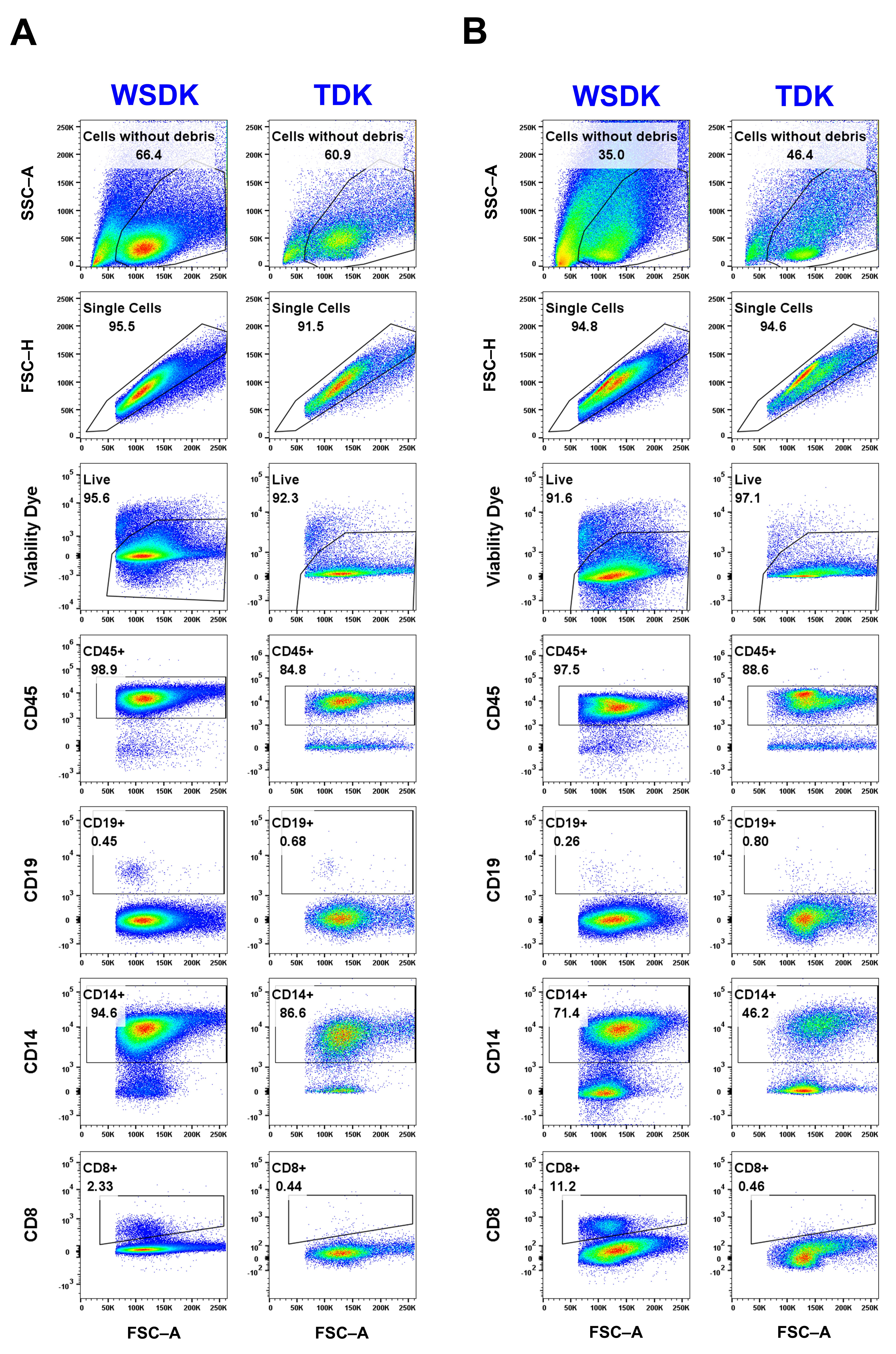

Supplement: Supplementary file 1 [file cancers-16-03942-s001.zip › SFig6.tif]

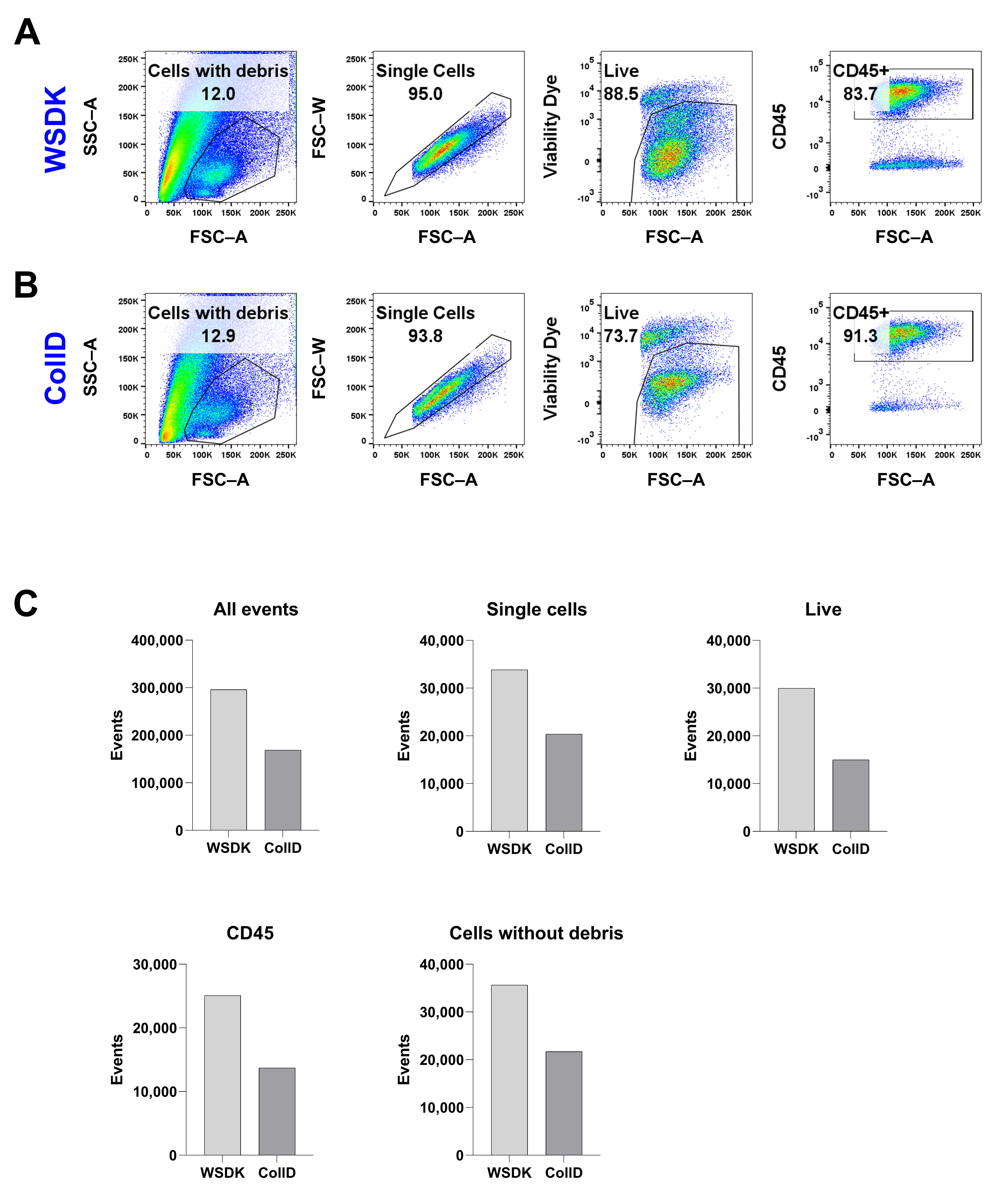

Supplement: Supplementary file 1 [file cancers-16-03942-s001.zip › SFig7.tif]
